# Priming Emotional Salience Reveals the Role of Episodic Memory and Task Conflict in the Non-color Word Stroop Task

**DOI:** 10.3389/fpsyg.2019.01826

**Published:** 2019-08-07

**Authors:** Chiao Wei Hsieh, Dinkar Sharma

**Affiliations:** School of Psychology, University of Kent, Canterbury, United Kingdom

**Keywords:** emotional stroop interference, task conflict, proactive control, reactive control, reversed sequential modulation, priming effect, anxiety

## Abstract

Previous research attempted to account for the emotional Stroop effect based on connectionist models of the Stroop task that implicate conflict in the output layer as the underlying mechanism (e.g., Williams et al., 1996). Based on Kalanthroff et al.’s (2015) proactive-control/task-conflict (PC-TC) model, our study argues that the interference from non-color words (neutral and negative words) is due to task conflict. Using a study-test procedure 120 participants (59 high and 61 low trait anxiety) studied negative and neutral control words prior to being tested on a color responding task that included studied and unstudied words. The results for the low anxiety group show no emotional Stroop effect, but do demonstrate the slowdown in response latencies to a block of studied and unstudied words compared to a block of unstudied words. In contrast, the high anxiety group shows (a) an emotional Stroop effect but only for studied negative words and (b) a reversed sequential modulation in which studied negative words slowed down the color-responding of studied negative words on the next trial. We consider how these findings can be incorporated into the PC-TC model and suggest the interacting role of trait anxiety, episodic memory, and emotional salience driving attention that is based on task conflict.

## Introduction

The Stroop task is often used to investigate executive control processes. In particular, to examine the ability to selectively attend to relevant and ignore irrelevant information (Stroop, 1935). The most common form of the task is one in which a word is printed in an ink color, with the focus to report the ink color and ignore the word. Typically, with color words the word and ink color can be congruent (e.g., word RED printed in red) or incongruent (e.g., word GREEN printed in red), with the difference in reaction time (RT) used to measure the Stroop effect. A neutral control (e.g., XXXX printed in red) can also be used to separate the Stroop effect into interference (difference between incongruent and neutral trials) and facilitation (difference between congruent and neutral trials) effects (MacLeod, 1991).

The Stroop task is thought to result from two types of conflict, informational conflict, and task conflict. Informational conflict is thought to be dependent on the congruency between the word and ink color, with conflict arising when the meaning of the word, and the ink color contradict each other (Klein, 1964; though see Shichel and Tzelgov, 2018 for further decomposition of informational conflict). Task conflict occurs between two potentially competing tasks. This can occur when certain stimuli become associated with certain tasks. For example, words tend to activate reading processes which results in competition between the task of reading and responding to the ink color (MacLeod and MacDonald, 2000; Goldfarb and Henik, 2007; Kalanthroff et al., 2013a, 2013b, Entel and Tzelgov, 2018; Sharma, 2018).

**FIGURE 1:**
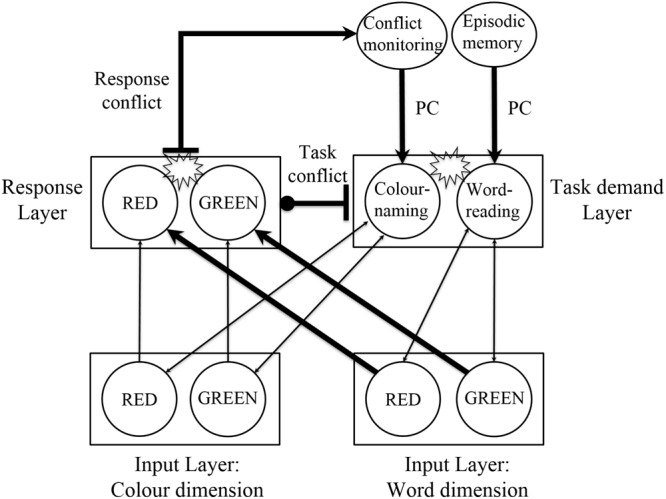
Proactive-control/task-conflict (PC-TC) model. Adapted from Kalanthroff et al. (2015). The task demand units are modulated by proactive control through the conflict monitoring node. The episodic memory node was not part of the PC-TC model and has been added to explain the current findings and those of Sharma (2018).

Connectionist models have been used to develop theoretical accounts of the Stroop effect (Cohen et al., 1990; Botvinick et al., 2001). Central to these models is the flow of information from an input layer (color and word units) to an output layer (color response units). In addition, a task demand layer (color naming and word reading units) is included to bias information flow based on task goals (e.g., instructions to focus on color naming) between the input and output layers. In such models, informational conflict results from competition between the output units (referred to as response conflict). Although early models relied on information flow in a bottom-up fashion, later models also allowed for a proactive top-down control mechanism (Botvinick et al., 2001; De Pisapia and Braver, 2006; Braver, 2012) to help maintain focus on the task goal. One source of evidence to support a proactive mechanism of control is the sequential modulation effect (aka the Gratton effect), in which incongruent trials are responded to faster when their previous trials are also incongruent than when they are congruent (Gratton et al., 1992; Kerns et al., 2004). It is thought that the attentional system monitors the degree of response conflict (a conflict monitoring node), and uses this to proactively increase the activation to the task goal of color naming to help reduce interference from words on subsequent trials (Botvinick et al., 2001). It is thought that the anterior cingulate cortex (ACC) is involved in the conflict monitoring mechanism (Botvinick et al., 2001). A more recent model, the Proactive-control/task conflict (PC-TC) model (Kalanthroff et al., 2015, 2018), inherits the response conflict mechanism from earlier models, but in addition includes a mechanism for task conflict. Kalanthroff et al. (2015, 2018) suggested that task conflict arises from the inhibitory connection between the task demand layer and the output layer (implemented by raising the response threshold for all the units in the output layer), where the level of inhibition is determined by the level of competition between the task demand (color naming and word reading units) units (see Figure 1).

Support for the PC-TC model comes from several sources. First, the reversed facilitation effect in which congruent words take longer to respond to than non-words under low PC (for a review see Kalanthroff et al., 2018). Here it is thought that the word reading task demand unit is activated by the congruent word in a bottom-up fashion to produce greater task conflict with color naming, compared to a non-word. Second, Sharma (2018) also provided evidence for the influence of task conflict using the non-color word Stroop task. Sharma used a priming procedure in which participants learned neutral words during a study phase (see also MacLeod, 1996). A subsequent testing session included two types of blocks. A block of unstudied words and a mixed block of studied and unstudied words. In both testing blocks the task was to ignore the words and respond to the ink color. Primed words resulted in slower responses to all studied and unstudied words in the mixed block compared to the unstudied block. Sharma suggested that the PC-TC model could explain this finding by assuming an episodic memory unit that holds the studied words temporarily and activates the word reading task demand unit, which can result in task conflict (see Figure 1). In addition, Sharma showed that in the second half of the mixed block, when presumably PC diminishes, there was a reversed sequential modulation in which studied words had longer latencies when preceded by studied words, compared to when preceded by unstudied words (for a similar finding with studied non-words see Dumay et al., 2018). This is consistent with a task conflict explanation that is due to reactive control from studied words.

Although much of the research using the Stroop task has focused on using color words, there is considerable evidence that non-color words can also slow down response latencies (Klein, 1964; Sharma and McKenna, 1998; Burt, 2002). One of the most common non-color word versions of the Stroop task is one in which negative emotional words are compared to neutral words, often labeled the emotional Stroop task (Williams et al., 1996; Algom et al., 2004; McKenna and Sharma, 2004; Dalgleish, 2005). The emotional Stroop task has been widely used to investigate attentional bias in anxiety and other emotional disorders such as depression, phobias, post-traumatic stress disorder (PTSD), obsessive-compulsive disorder, and panic disorder. The difference in response latencies between negative emotional and neutral words is referred to as the emotional Stroop effect. Findings suggest that both non-clinical individuals with high trait anxiety and clinically anxious individuals show attentional bias toward threat-related words, whereas such threat-related bias is not found in non-anxious individuals (Bar-Haim et al., 2007; Phaf and Kan, 2007; Yiend, 2010).

Following the connectionist model of Cohen et al. (1990), previous models to explain the emotional Stroop effect have tacitly assumed that emotional interference occurs at the output layer due to response conflict. Williams et al. (1996) hypothesized that input units which represent negative emotional words could have higher resting activation levels (implemented by regulating the gain parameter). Consequently, the greater activation throughout the negative emotional word processing pathway results in greater competition with color response units in the output layer. Matthews and Harley (1996) hypothesized that attentional bias is contingent on the allocation of voluntary attention to threat stimuli. Adapting from Cohen et al.’s (1990) model, they introduced a threat monitoring unit in the task demand layer (to simulate trait like effects), as well as an emotion word unit in the input layer. When a threatening word is presented, the active threat monitoring task demand unit would sensitize the threat emotional word processing pathway which would result in greater response conflict in the output layer. An alternative model for negative emotional interference was provided by Wyble et al. (2008), who suggested that negative words affected the balance of control proactively. This was implemented by mutual inhibition between the conflict monitoring unit and the negative emotional unit in their adaptive attentional control layer, and supports the conclusion that negative emotional words reduce proactive control to the task goal of color naming. This approach is also consistent with other more general models that make similar predictions, such as the Dual Competition Model (Pessoa, 2009) and the attentional control theory (Eysenck et al., 2007).

Since the role of task conflict has not been considered in connectionist models of the emotional Stroop effect, here we consider how this might be implemented. In the PC-TC model this can occur in a number of ways, but one way might be by greater activation of the word reading task demand unit. The word reading task demand unit can be activated in two ways, either in a bottom-up reactive fashion (e.g., by activation from negative word input units) or in a top-down proactive control mechanism (e.g., by a threat monitoring task demand unit in high anxious individuals or more generally by priming from negative schemas) that enables the word reading task demand unit to compete with the color naming task demand unit. Evidence for both mechanisms was provided by Sharma (2018) when comparing trials within and between blocks. Between blocks proactive control was evidenced as a general slowdown, in particular the neutral words in the block containing studied words were slower than those in a block without studied words. On the other hand, within a block of studied and unstudied words, an indication of reactive control came from a reversed sequential modulation in which studied words were slower to respond to when preceded by another studied word than an unstudied word.

The main aim of our research was to use the priming procedure developed by Sharma (2018) to investigate further evidence for the role of task conflict in the non-color word Stroop task. In our experiment participants study both negative and neutral words during an initial study phase, which is followed by a test phase comprising four blocks with different word categories: (1) a block of unstudied neutral words [C]; (2) a block of unstudied negative and neutral words, [NC]; (3) a mixed block of studied and unstudied neutral words, [CsC]; and (4) a mixed block of studied negative and unstudied neutral words, [NsC]. This leads to seven word categories, which are represented by the following labels: (note that letters within square brackets refer to the type of block and letters outside the square brackets refer to the type of word) [C]-C, [NC]-C, [NC]-N, [CsC]-C, [CsC]-Cs, [NsC]-C, and [NsC]-Ns. As previous research highlights differential results for high and low anxiety with negative emotional stimuli, we also investigate the role of trait anxiety (Kalanthroff et al., 2016).

We expected to replicate Sharma’s (2018) finding of a general slowdown for the studied [CsC] compared to the unstudied neutral words [C] that is an indicator of task conflict from proactive control. We also extend this research to using studied negative words and expected to find a similar general slowdown for a [NsC] block compared to the unstudied [C] block.

If there is a general hypervigilance for negative stimuli in high anxiety, then this may appear either as longer response times for negative words than neutral words in [NC] or [NsC], and/or as a general slowing in block [NC] or [NsC] compared to [C]. However, previous research on mixing negative and neutral words has shown weak effects (Williams et al., 1996). Indeed there is strong evidence that priming plays an important role in the emotional Stroop effect (Richards et al., 1992; Holle et al., 1997; Lundh and Czyzykow-Czarnocka, 2001). For example, Richards et al. (1992) showed that high anxious participants do not show an emotional Stroop effect when neutral and negative words were randomly mixed. However, a more robust effect occurred after negative mood induction or when negative and neutral words were blocked during the test (see also Holle et al., 1997). Priming the anxiety schema prior to testing can also have similar effects (see Lundh and Czyzykow-Czarnocka, 2001). This suggests that negative words produce interference in high anxiety but only when they have been primed. In line with Richards et al. (1992) we expected to find an emotional Stroop effect for high anxious participants in the block containing studied negative words, [NsC]. Comparing the neutral words in the [NsC] block and the [C] block could help to distinguish between response conflict and task conflict. The general prediction is that if negative stimuli increase response conflict, then response latencies will speed up across trials due to the feedback from conflict monitoring increasing activation of the color naming task demand unit (Botvinick et al., 2001). If negative words increase activation of the word reading task demand unit, then the PC-TC model would predict a slower response to neutral words in the [NsC] block than the [C] block.

In line with Attentional Control Theory, we also expected there to be a reduced effect of proactive control in high trait anxiety (Eysenck et al., 2007; Berggren and Derakshan, 2013; Kalanthroff et al., 2016). A reduced proactive control could be seen as a general slowdown from studied words that is larger in the low anxiety group than the high anxiety group. In addition, it suggests that further analysis of the mixed blocks may show signs of reactive control that is more apparent in the high anxiety group than the low anxiety group. In particular we contrasted pairs of consecutively presented trials: CsCs or NsNs trials with CCs or CNs trials, respectively. If the effects of reactive control are due to response conflict, then the PC-TC model predicts a sequential modulation effect in which studied words are faster to respond to after studied words. However, as shown by Sharma (2018), if the effects of reactive control lead to task conflict, then the PC-TC model predicts a reversed sequential modulation effect: slower responses to studied words on the trial after a studied word.

## Materials and Methods

### Participants

A 120 native English-speaking students from the University of Kent took part in this study for course credits or 5 pounds in cash. The sample comprised of 104 females and 16 males, aged 18–49, and mean age of 20.72 (*SD* = 4.755). Ethical approval was given by the School of Psychology Ethics committee at the University of Kent.

### Design

A 7 × 2 mixed factorial design was employed. Word category ([C]-C, [NC]-C, [NC]-N, [CsC]-C, [CsC]-Cs, [NsC]-C, and [NsC]-Ns) was the within-subject factor, and Trait group (high, low) was the between-subject factor. The dependent variable was the mean correct response latency to respond to the words.

### Apparatus and Materials

The experiment program was written in Psychopy 1.83.04 and presented on a 21-inch Dell^®^ widescreen monitor. RT was measured during the Stroop tasks. The manual responses, presentation, and randomization of the words were controlled by Psychopy 1.83.04. The words used are shown in Table 1.

**TABLE 1:** Word lists used in the study.

**Negative**	**Negative**	**Control**	**Control**	**Control**	**Control**	**Control**	**Control**
fear	pain	area	card	pipe	game	hall	tool
hate	lose	limb	poem	unit	path	rock	deep
shun	thug	soar	trot	whiz	claw	raft	meat
angry	argue	chair	sugar	wheel	paint	hotel	queen
crime	abuse	plant	stage	river	metal	mouth	union
death	sorry	board	voice	class	press	title	month
horror	danger	enable	launch	expand	import	formal	phrase
cancer	threat	belief	bottle	bridge	custom	square	manage
betray	maggot	mascot	turret	tendon	wobble	cortex	pebble
anxiety	awkward	harvest	surgeon	whistle	surname	reactor	outlook
corrupt	illness	cartoon	lottery	texture	vaccine	predict	observe
selfish	hostile	tourist	sticker	shelter	pursuit	thermal	booklet
suicide	violent	segment	profile	prepare	academy	kitchen	formula
horrible	disaster	revision	retrieve	clinical	estimate	adequate	abstract
arrogant	massacre	mainland	activate	reminder	altitude	shipment	shepherd
nuisance	stubborn	tangible	teaspoon	molecule	landmark	nutshell	homeland
depressed	terrorist	voluntary	physician	librarian	diagnosis	ancestral	alignment
miserable	obnoxious	peninsula	machinery	offspring	geography	crossover	astrology
disgusting	frustrated	moderation	elementary	coordinate	adjustment	inevitable	convincing
suspicious	disability	subscriber	occupation	projection	calculator	curriculum	researcher

A total of 40 negative emotional words were chosen from Affective Norms for English Words (Warriner et al., 2013) and separated into two sets of 20 words. 120 neutral words were selected from the English Lexicon Project (Balota et al., 2007) and divided into six sets of 20 words. Each set contained an equal number of 4, 5, 6, 7, 8, 9, and 10 letter words, which were matched for word frequency (average Log frequency HAL of 8.84), which was in the midrange for the corpus of words (Range 0–17) (Balota et al., 2007); word valence (average valence mean of 2.56 and 5.59 for negative emotional and neutral words, respectively) (Range 1.26–8.53), and word arousal (average arousal mean of 5.52 and 3.87 for negative emotional and neutral words, respectively) (Range 1.6–7.79) (Warriner et al., 2013).

### Procedure

An information sheet and a consent form were given to each participant upon their arrival. After signing the consent form, participants sat in front of the pc monitor and were asked to read through the instructions for the experiment’s procedure. There were four phases in this study: the study phase, test phase, recall phase, and questionnaire phase.

#### Study Phase

Each participant was shown 40 words in white print on a black background, which mixed 20 negative emotional words from one of two negative emotional word sets and 20 control words from one of six neutral word sets, and was asked to memorize them as best as they can. To help participants enhance their memory, after a word was shown a five-point grading scale was presented (1 = 0%, 2 = 25%, 3 = 50%, 4 = 75%, and 5 = 100%), in which they rated how strong the word related to themselves. Each word was presented one at a time in white print at the center of the screen for 1500 ms, followed by an 800 ms blank screen prior to the five-point grading scale. The grading stage remained until a response was given before the next word was shown.

#### Test Phase

Practice trials were provided before the experimental Stroop task, which consisted of 20 non-words (e.g., dfbvxz, whcag, and vfjtd). These 20 non-words were printed in each of four colors (red, green, blue, and yellow) on a black background for 80 trials which were randomly displayed. Each trial started with a 1000 ms fixation at the center of the screen, followed by a non-word which remained until a response was provided before the next trial started. Participants were instructed to place their index and middle fingers from each hand on top of four keys (z = red, x = green, n = blue, and m = yellow) on a QWERTY keyboard, and they were asked to ignore the non-words and respond to the ink color as quickly and as accurately as possible.

The general instructions and procedure for the experimental Stroop task were identical to the practice phase. There were four blocks ([C], [NC], [CsC], and [NsC]) with two sets of words, comprised of either two sets of 20 control words or 20 control words mixed with 20 negative words. In each block, 40 words were printed in each of four colors for 160 trials, resulting in 640 trials for the Stroop task. The two sets of negative emotional words and six sets of neutral control words were assigned to four experimental blocks and counterbalanced across participants. Each word was presented in a random order in each block.

As soon as a block was completed, participants were given an option to take a short break and were instructed to carry on with the next block by pressing the space bar. The order of four blocks was counterbalanced across participants.

#### Recall Phase

The test phase was followed by the recall phase, in which participants had 180 s to write down as many words that they had seen during the study phase as they could remember on a blank sheet of paper.

#### Questionnaire Phase

The questionnaire phase followed the recall phase. The Spielberger State-Trait Anxiety Inventory (STAI) was given to participants, consisting of 20 statements for state anxiety which indicates how you feel right now, and trait anxiety implying how you feel in general, respectively (Spielberger et al., 1983).

## Results

### Analysis of the Stroop Task

#### Data Preparation

Scores on the STAI-trait ranged from 20 to 78 (*M* = 48.80, *SD* = 12.33). Based on norms collected between 2005 and 2007 (*N* = 368) from students at University of Kent, trait anxiety scores of 50 or above represent percentile ranks 75% [85% (for males) and 72% (for females)]. Participants were assigned to the low (<50) or high (>=50) trait anxiety group for the ANOVA analysis. Average STAI-trait score in the high anxiety group (range 50–78, *M* = 58.56, *SD* = 7.39, *N* = 59) low anxiety group (range 20–49, *M* = 39.36, *SD* = 8.02, *N* = 61).

Four participants’ data were removed: one was due to a high error rate (18.9%) and the other three data due to long RTs (above 2.5 standard deviation). The error rate of the remaining 116 participants (Low trait: *N* = 59; High trait: *N* = 57) was 4.50%. Prior to the analysis of mean correct response latencies, the first trial of each block and trials with an RT less than 200 ms and larger than 3,000 ms, which was 5.5% of the trials, were excluded.

#### Analysis of Response Latencies

The first analysis was executed on the mean correct RTs, using a 7 × 2 two-way mixed analysis of variance (ANOVA), with Word category ([C]-C, [NC]-C, [NC]-N, [CsC]-C, [CsC]-Cs, [NsC]-C, and [NsC]-Ns) as a within-subject factor, and Trait group (high, low) as a between-subject factor. Greenhouse-Geisser corrected values were reported when the sphericity assumption was violated.

**FIGURE 2:**
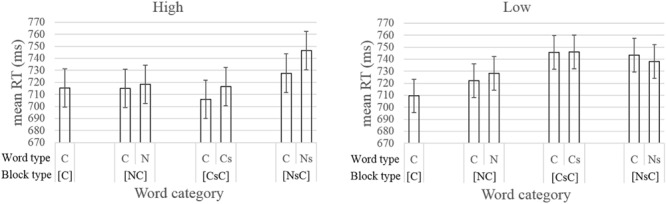
Mean correct reaction times for high and low trait anxiety group in each Word category. Error bars represent the 95% confidence interval adjusted for the within-subject design, calculated separately for high and low trait anxiety group (Masson and Loftus, 2003). C, neutral word; N, negative word; s, word is studied.

The analysis revealed a significant main effect of Word category, *F*(3.29,375.18) = 3.59, MSe = 6133.02, *p* = 0.011, ηp^2^ = 0.031. Bonferroni corrected *t*-tests indicated that there was a significant difference between [NsC]-Ns (M = 742.25 ms, SE = 14.17) and [C]-C (*M* = 712.37 ms, SE = 12.41) words (*p* = 0.007). A main effect of Trait group was not significant *F*(1,114) = 0.277, MSe = 114719,80 *p* = 0.600, ηp^2^ = 0.002. However, there was an interaction between Word category and Trait group, *F*(3.29,375.18) = 2.67, MSe = 6133.02, *p* = 0.042, ηp^2^ = 0.023. Bonferroni corrected *t*-tests indicated that in the low trait anxiety group, [CsC]-C (*M* = 745.62ms, *p* = 0.004), [CsC]-Cs (*M* = 745.96 ms, *p* = 0.003), [NsC]-C (*M* = 743.45ms, *p* = 0.025) words took longer to respond to than the [C]-C (*M* = 709.42 ms) words. On the other hand, in the high trait anxiety group, the [NsC]-Ns (*M* = 746.40 ms, *p* = 0.003) words took longer than [CsC]-C (*M* = 705.76 ms) words (see Figure 2).

Further analysis of this interaction involved planned comparisons. First, there was an emotional Stroop effect for high trait anxiety with studied negative words, *F*(1,56) = 8.49, *p* = 0.006, ηp^2^ = 0.13, but not unstudied negative words, *F*(1,56) = 0.45, *p* = 0.51, ηp^2^ = 0.008. There was no emotional Stroop effect for low trait anxiety (both *F*’s < 1, *p*’s > 0.37). Second, we looked for evidence for proactive task conflict across the blocks. For each trait group we asked if the mixed blocks took longer than the baseline block [C]. For the low anxiety group this was significant ([C] vs. [CsC], *F*(1,58) = 18.86, *p* < 0.001, ηp^2^ = 0.25; [C] vs. [NsC], *F*(1,58) = 10.44, *p* = 0.002, ηp^2^ = 0.15). This replicates similar findings by Sharma using neutral words and extends these to studied negative words. For the high trait anxiety group this was not significant for [NsC] vs. [C], F(1,56) = 3.19, *p* = 0.08, ηp^2^ = 0.05, or [CsC] vs. [C], *F*(1,56) = 0.12, *p* = 0.73, ηp^2^ < 0.01, or [NC] vs. [C], *F*(1,56) = 0.61, *p* = 0.4, ηp^2^ = 0.01. These findings suggest that in high trait anxiety, studied words tend not to slow latencies for blocks with studied words. The above results generally indicate that blocks with studied words tend to have longer latencies than a block of unstudied control words, and that this seems to reduce with trait anxiety. Correlations with trait anxiety scores, however, showed that this impression was only supported for [CsC] [*r*(114) = −0.22, *p* = 0.016)] but not [NsC] [*r*(114) = −0.019, *p* = 0.84].

**FIGURE 3:**
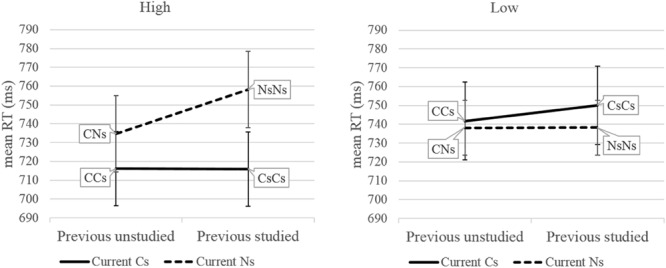
Showing the reversed sequential modulation effects within block [CsC] (trial CsCs vs. trial CCs) and block [NsC] (trial NsNs vs. trial CNs) for high and low trait anxiety group. Error bars represent 95% confidence interval for the paired difference between two means, computed separately for the effects within block [CsC] and block [NsC] (Pfister and Janczyk, 2013). C, neutral word; N, negative word; s, word is studied.

To investigate whether priming words results in task conflict from reactive control we carried out a series of planned comparisons within the two mixed blocks. We asked whether studied words take longer to respond to when preceded by studied words compared to unstudied words (i.e., trial CsCs vs. trial CCs or trial NsNs vs. trial CNs). For the low anxiety group there was no significant reversed sequential modulation effect in either [CsC], *t*(58) = 0.81, *p* = 0.42 or [NsC], *t*(58) = 0.002, *p* = 0.99. For the high anxiety group there was a significant reversed sequential modulation effect in [NsC], *t*(56) = 2.31, *p* = 0.025 but not [CsC], *t*(56) = 0.02, *p* = 0.98 (see Figure 3). The modulation found in high anxiety for studied negative words suggests that the reversed sequential modulation increases with higher levels of trait anxiety. This was supported by a positive correlation between trait anxiety scores and reversed sequential modulation scores in the [NsC] block, *r*(114) = 0.187, *p* = 0.04. The correlation between trait anxiety and reversed sequential modulation scores in the [CsC] block was not significant, *r*(114) = −0.155, *p* = 0.097, though the negative direction indicates that lower anxiety may be associated with a reversed sequential modulation effect from studied neutral words.

### Analysis of Recall Phase

Prior to the analysis, the words written down by participants during the recall phase were checked. Misspellings were accepted (e.g., masaccare for massacre) but the altered forms were excluded (e.g., angry changed to anger).

**FIGURE 4:**
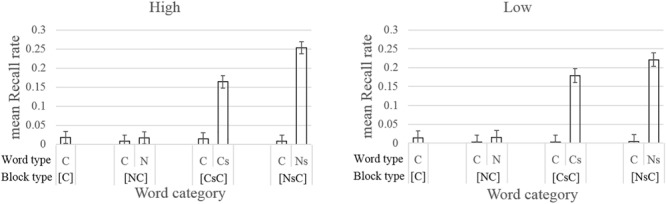
Mean recall rate for high and low trait anxiety group in each Word category. Error bars represent 95% confidence interval adjusted for the within-subject design, calculated separately for high and low trait anxiety group (Masson and Loftus, 2003). C, neutral word; N, negative word; s, word is studied.

A 7 × 2 mixed ANOVA was conducted with Word category ([C]-C, [NC]-C, [NC]-N, [CsC]-C, [CsC]-Cs, [NsC]-C, [NsC]-Ns) as a within-subject factor, and Trait group (high, low) as a between-subject factor. The results revealed a significant main effect for Word category *F*(2.37, 270.63) = 239.44, MSe = 4.57, *p* < 0.001, ηp^2^ = 0.68 but not for Trait group *F*(1,114) = 1.34, MSe = 2.34, *p* = 0.249, ηp^2^ = 0.01 or the Word category × Trait group interaction, *F*(2.37,270.63) = 1.25, MSe = 4.57, *p* = 0.289, ηp^2^ = 0.01. Mean recall rates for studied words [NsC]-Ns (*M* = 0.24) and [CsC]-Cs (*M* = 0.17) are significantly higher than other word categories, all *t*’s > 12.85, *p*’s < 0.01 (see Figure 4). Moreover, mean recall was significantly higher for [NsC]-Ns (*M* = 0.24) than [CsC]-Cs (*M* = 0.17), *t*(115) = 4.76, *p* < 0.001. We also checked if the difference between [NsC]-Ns and [CsC]-Cs correlated with trait anxiety scores; it did not, *r*(114) = 0.120, *p* = 0.199.

We also note that the results were the same when analyzed using the lenient criteria in which altered forms were accepted as well (only the main effect of Word category was significant *F*(2.41,274.19) = 252.55, MSe = 4.60, *p* < 0.001, ηp^2^ = 0.69; all other main and interaction effects were not significant *F*’s < 2.2, *p*’s > 0.1).

## Discussion

The memory results were as expected: (a) higher recall for studied words than unstudied words. (b) Studied negative words have higher recall than studied neutral words. (c) No interaction with trait anxiety. As expected, these results show the typical episodic memory advantage for recently attended words and words that are semantically related. The lack of interaction with trait anxiety is consistent with previous reviews of the memory bias literature (see Williams et al., 1997; Mitte, 2008). There is some evidence that a memory bias with trait anxiety can occur for free recall memory tasks but only when the depth of processing is shallow during the study phase (for a review see Herrera et al., 2017). Our findings are consistent with these reviews, as a high level of processing (words were rated for self-relevance) was required during the study phase.

The main findings, however, are from the response latencies to the non-color (neutral and negative emotional) words. For the low anxiety group, there are two key findings. First, neutral words in the studied block [CsC] took longer to respond to than neutral words in the unstudied block [C]. This evidence is consistent with the task conflict hypothesis that is driven by proactive control and replicates findings by Sharma (2018) for studied neutral words. Within the PC-TC model, this could be due to stronger proactive activation of the word reading task demand unit in studied blocks. Second, the slowdown for studied neutral words also generalizes to a block with studied negative words (i.e., [NsC]), and therefore suggests that negative words can also slow down responses in low anxiety but only when these words have been primed. As there was no difference between the two studied (neutral and negative) blocks, together these two findings highlight the influence of studying words in the non-color Stroop task. Therefore, this extends the original work of MacLeod (1996) and replicates the findings by Sharma (2018) to further demonstrate that the study-test methodology can be used to investigate implicit memory in the non-color word Stroop task.

For the high trait anxious group, there are three main findings. First, an emotional Stroop effect in the [NsC] block but not in the [NC] block. This supports previous research that priming a negative scheme (in our study by learning negative and neutral words during an initial study phase) can generate attentional biases (c.f. Richards et al., 1992; Holle et al., 1997; Lundh and Czyzykow-Czarnocka, 2001). In our study, the priming was specific to negative words for the high trait anxiety group and replicates the findings by Richards et al. (1992) and Holle et al. (1997) where negative words induced interference after negative mood induction or by presenting negative words in a single block of trials. More generally this finding also implicates the importance of memory processes when considering interference in the non-color word Stroop task. For example, it is possible that the priming effects found for studied negative words in high anxiety may have activated episodic memory (see Figure 1). In addition, it is possible that such memory activation also initiates higher thought processes such as rumination or self-reflective processes. This may also explain why studied neutral words did not show a similar effect in the high anxiety group. Further research is therefore required to further explore this possibility.

Second, although the high trait anxious group showed an emotional Stroop effect in the [NsC] block, there was no evidence of a general slowdown for the neutral words in the [NsC] block compared to the baseline [C] block. The lack of a general slowdown contrasts with the slowdown seen for the low anxiety group. This finding is consistent with Attentional Control Theory which suggests that in high trait anxiety the balance of control shifts away from proactive control. In the PC-TC model this could be implemented as a reduced top-down activation of the word reading task demand unit.

Third, in high trait anxiety, studied negative words took longer to respond to when preceded by studied negative words compared to unstudied neutral words. Here, we speculate on several potential explanations for the reversed sequential modulation. Sharma (2018) reported a similar finding with studied neutral words, namely a reversed sequential modulation for studied neutral words. He suggested a possible reactive control mechanism that activates task conflict in the PC-TC model. A similar mechanism could be suggested for studied negative words in high trait anxiety. However, it is also possible to suggest the influence of a proactive control mechanism. In Figure 1, the word reading task demand node can be activated by proactive control from episodic memory. Although this influence may be weaker in high anxiety, our results suggest that the episodic memory unit may be activated when responses are made to two consecutively presented studied negative words. These two suggestions point to task conflict as a potential mechanism. However, it is also possible to suggest that task conflict is not involved if it is assumed that two consecutively presented studied negative words require greater attentional resources that subsequently results in a relaxation of cognitive control, as suggested by the Duel Competition Model (Pessoa, 2009). In a connectionist model without task conflict, this could be implemented by inhibition of the conflict monitoring unit analogous to the inhibition from the negative emotion unit in the Adaptive Attentional Control model (Wyble et al., 2008). If this was the case, then more detailed predictions from the Wyble et al. (2008) model would suggest that studied words slow down subsequent neutral trials analogous to the slow effect reported by McKenna and Sharma (2004) for negative stimuli. We checked for a slow effect from studied words (negative or neutral), but could not find any evidence. Future research could examine the conditions under which slow effects appear. However, we believe the current work is more parsimonious with a model that includes task conflict.

Two puzzling features of our results suggest further avenues for future research. First, we did not find a reversed sequential modulation for studied words in the low anxiety group. This did not replicate the reversed sequential modulation for studied neutral words found by Sharma (2018). We suggest this may be due to the stronger proactive control from episodic memory to the word reading task demand unit in our study than in Sharma. This may be due to using a larger set of studied words (40 in our experiment compared to 20 in Sharma), and/or using negative words which forms a stronger semantic category than the neutral words set. Second, for the high anxiety group the reversed sequential modulation did not occur for the studied neutral words. This is surprising, particularly as it is thought that in high anxiety the balance of control shifts toward reactive control. One explanation might be that using a larger studied word set may have reduced the saliency of each individual item. However, for the studied negative words their stronger semantic associations may have enabled them to maintain a stronger level of priming.

In conclusion, our findings provide further evidence in support of using the priming technique to elucidate the role of task conflict in the non-color word Stroop task. For low anxiety, studying (neutral and negative) words resulted in a general slowdown that was attributed to task conflict resulting from a proactive control mechanism that increases activation of the word reading task demand node. For high anxiety, the general slowdown is limited suggesting a reduced influence from proactive control.

## Data Availability

The raw data supporting the conclusions of this manuscript will be made available by the authors, without undue reservation, to any qualified researcher.

## Author Contributions

CWH and DS designed the study, analyzed the data, and wrote the manuscript. CWH collected and cleaned the data.

## Conflict of Interest Statement

The authors declare that the research was conducted in the absence of any commercial or financial relationships that could be construed as a potential conflict of interest.
